# The protocol for a phase 2A, multi centre, double-blind, randomized, placebo-controlled, parallel-group study to assess the effect of 12 weeks treatment with oral PXS-4728A on microglia activation, as measured by positron emission tomography, in participants with isolated rapid eye movement sleep behaviour disorder

**DOI:** 10.3389/fneur.2026.1951452

**Published:** 2026-10-28

**Authors:** Karolien Groenewald, Johannes C. Klein, Tim D. Fryer, Graham Smith, Nicolas Dzamko, Michelle Chua, Andrew M. Scott, Jana Baskar, Dieter Hamprecht, Jacqui Everett, Joanna Leadbetter, Michele T. Hu, Simon J. G. Lewis

**Affiliations:** 1 Nuffield Department of Clinical Neurosciences and Oxford Parkinson’s Disease Centre, University of Oxford, Oxford, United Kingdom; 2 Wolfson Brain Imaging Centre, University of Cambridge, Cambridge, United Kingdom; 3 Faculty of Medicine, Health and Human Sciences, Macquarie University, Sydney, NSW, Australia; 4 Austin Health, University of Melbourne, Melbourne, VIC, Australia; 5 Olivia Newton-John Cancer Research Institute, La Trobe University, Melbourne, VIC, Australia; 6 Syntara Limited, Frenchs Forest, NSW, Australia

**Keywords:** clinical trial protocols as topic, microglia, PET imaging, REM sleep behaviour disorder, synucleinopathies

## Abstract

**Introduction:**

Isolated REM-sleep behaviour disorder (iRBD) is a well-described prodromal feature of synuclein diseases, including Parkinson’s disease (PD), dementia with Lewy bodies and, less frequently, multiple system atrophy. Neuroinflammation has been reported in iRBD and is potentially a major contributor to synuclein accumulation and propagation. Positron emission tomography (PET) imaging of the translocator protein (TSPO) overexpressed by activated microglia has been used to demonstrate neuroinflammation in both iRBD and manifest PD. Although immune-modulating therapies have not shown direct benefit in PD, there are to date no reported trials in prodromal disease. This paper describes the protocol for the first immune-modulating drug trial in iRBD, which could provide evidence for an early neuroprotective intervention strategy in prodromal PD.

**Methods and analysis:**

This is a phase IIa, randomised, double-blind, placebo-controlled, parallel-group trial of the small molecule PXS-4728A in iRBD. Forty participants with definitive iRBD, one or more features of Parkinsonism, impaired olfaction and/or impaired colour vision will be randomised 3:1 (treatment: placebo) for a 12-week treatment period, followed by a 12-week washout. The primary outcome will assess the effect of PXS-4728A on neuroinflammation, measured by PET using the TSPO radioligand [^11^C]-PK11195 across nigro-striato-cortical regions in the treatment arm (within-arm) from baseline to week 12. Between-arm differences will be treated as exploratory. Drug safety and tolerability will also be assessed as a primary outcome. Secondary outcomes include further regional and whole-brain analysis of [^11^C]-PK11195 binding. Exploratory outcomes will assess change in clinical scales of prodromal symptoms (motor and non-motor), biological markers from blood and CSF, and digital biomarkers (actigraphy, motor and cognitive).

**Study protocol registration:**

https://clinicaltrials.gov/study/NCT05904717; Identifier, NCT05904717.

## Introduction

Isolated Rapid Eye Movement Sleep Behaviour Disorder (iRBD) is a parasomnia and recognised prodrome of synucleinopathies, including Parkinson’s disease (PD), dementia with Lewy bodies (DLB) and less commonly, multiple system atrophy (MSA) (1, 2). Currently, iRBD is defined by the ICSD-3-TR criteria as: repeated episodes of vocalisations and/or complex motor behaviours, documented to occur during REM-sleep or presumed to occur during REM sleep based on clinical history of dream enactment behaviours (DEB’s), polysomnographic (PSG) evidence of REM sleep without atonia (RSWA) and no other disturbance causing the disorder (mental, sleep, medication or substances) (1). RSWA with subsequent DEB’s are related to dysfunction of the sublaterodorsal nucleus in the brainstem, which exerts spinal motor neuron inhibition during normal REM-sleep. It is reported that iRBD is prevalent in 1% of people above the age of 60 years (3, 4) but significantly, carries a conversion risk to synucleinopathy of 6% per year and over 15 years 80–90% of individuals will have converted to PD, DLB or MSA (5, 6). The prevalence of iRBD in PD is around 40–50% and the presence of this symptom has been associated with a more aggressive clinical subtype related to accelerated progression across motor and non-motor symptoms (7–11).

Patients with iRBD may have increasing motor and non-motor symptoms that fail to meet the threshold for a clinical diagnosis of a defined neurodegenerative disease (12). However, clinical features including hyposmia, reduced colour vision discrimination, autonomic dysfunction, mood and cognitive changes, as well as mild motor symptoms are present and can predict earlier transition to disease (13). Currently, iRBD is managed symptomatically with no curative treatment established. Sleep environment modification is recommended in iRBD to prevent the risk of injury during nocturnal events. Recommended medication for the symptomatic management of iRBD include melatonin, clonazepam and pramipexole (13, 14).

The pathophysiology occurring during the prodromal period in a subset of synuclein disease, including iRBD, has been described by the “body-first” hypothesis where it is proposed that misfolded α-synuclein aggregates originate from the enteric nervous system, with subsequent central spread via the autonomic nervous system (vagus nerve) leading to the neuropathological hallmark of PD, Lewy Body inclusions in the substantia nigra of the brainstem (15). This hypothesis has been replicated in animal studies, where α-synuclein aggregates spread from the enteric to central nervous system through the autonomic nervous system (16). Additionally, α-synuclein inclusions occur in the enteric nerve fibres prior to a diagnosis of PD (17). In a recent postmortem study of people with RBD, including three people with iRBD, early-stage α-synuclein aggregation in the brainstem and limbic system with associated degeneration was shown, further illustrating early synuclein propagation to the central nervous system occurring in prodromal disease (18).

Beyond the hallmark α-synuclein inclusions associated with the neurodegenerative disease spectrum, McGeer et al. first demonstrated activated microglia in postmortem tissue of people with PD in 1988 (19). In 2007, Su et al. demonstrated that α-synuclein inclusions activate microglia, leading to an inflammatory cascade, including a local increase in TNFα and reactive oxygen species (ROS). This work suggested that misfolded α-synuclein protein may be initiating a neuroinflammatory response, which likely contributes to nigrostriatal pathology (20). Postmortem studies have replicated evidence for this inflammatory cascade by showing increased levels of TNFα, interleukins (IL-1β, IL-6) and transforming growth factor β1 in the brain and ventricular cerebrospinal fluid in patients with PD (21–27). In the peripheral immune system, studies have also shown that there is an inflammatory dysregulation in blood monocytes, cytokines and T-lymphocyte populations in people with PD (28–31). Moreover, animal studies have demonstrated that LPS-induced reactive microglia can promote α-synuclein propagation (20) thereby driving a bidirectional pathophysiology.

Further evidence of neuroinflammation has been demonstrated using in-vivo translocator protein (TSPO) PET imaging. TSPO is a protein mainly expressed in the outer mitochondrial membrane, with overall low expression in healthy controls. However, its levels increase with glial (astrocytic and microglial) activation (32, 33). In this manuscript, “TSPO binding” refers to the measured [^11^C]-PK11195 binding potential, which we interpret as an *in vivo* marker of microglial activation, a principal component of neuroinflammation. Despite [^11^C]-PK11195 being a first-generation TSPO PET radioligand with limited central penetration and a relatively low signal-to-noise ratio, it remains widely used. This is due in part to the genetic variability in the TSPO gene (rs6971 polymorphism), which induces measurable non-affinity binding across the resultant affinity classes for second-generation ligands, including [^11^C]-PBR28, [^11^C]-DPA713, [^18^F]-FEPPA and [^18^F]-DPA714 (34, 35), but also because of the substantial amount of reference data already generated with [^11^C]-PK11195 (36–44). In 2005, Ouchi et al. showed increased binding of [^11^C]-PK11195 in the contra-lateral midbrain of the affected side in early, untreated PD when compared to healthy controls (36). These findings have been replicated in several other PET studies in manifest PD (38, 40, 41, 45–49). Additional regions of increased binding, albeit variable, included the substantia nigra, striatum, thalamus, cortical areas and cerebellum (50).

In iRBD, the first case–control study included 20 participants and showed increased [^11^C]-PK11195 binding in the substantia nigra, as measured by [^11^C]-PK11195, which was paired with reduced putaminal dopaminergic function as measured on [^18^F]-DOPA PET (41). A follow up report by the same group showed a greater reduction in nigrostriatal function on repeat [^18^F]-DOPA PET scanning after 3 years in iRBD subjects with elevated baseline [^11^C]-PK11195 binding (39). Another report by this group (with one new participant added), looking at monoaminergic dysfunction, showed additional significant increased [^11^C]-PK11195 binding in the occipital lobes of iRBD compared to control participants (40). A final paper by the same group investigating cortical cholinergic dysfunction and microglial activation in the substantia innominata in iRBD did not find group-level differences in [^11^C]-PK11195 binding between iRBD and controls. However, they did report a negative correlation between cortical cholinergic function (as measured by [^11^C]-donepezil PET) and [^11^C]-PK11195 binding in the substantia innominata (43).

Retrospective studies and meta-analyses have shown a decreased risk in populations using anti-inflammatory medications (non-steroidal, anti-IL and anti-TNF) for developing PD (51). An overview of anti-inflammatory and immunomodulatory drugs trialled in PD published in 2024, concluded that although no large trials have shown definitive benefit of these treatments in established PD, some early studies have shown promising efficacy (for example NLRP3 inflammasome inhibitors) (52). A need for earlier intervention may be one reason for a lack of efficacy to date, however there are no published data on the role of immunomodulating treatments in prodromal PD.

Therefore, iRBD represents an important window of opportunity for disease modification to slow down or interrupt progression to an established neurodegenerative disease (synucleinopathy). PXS-4728A is an oral, irreversible inhibitor of semicarbazide-sensitive amine oxidase (SSAO) and brain monoamine oxidase B (MAO-B). PXS-4728A (previously BI 1467335) has been studied pre-clinically and was well tolerated in multiple clinical Phase 1 and 2 trials targeting diabetic retinopathy and non-alcoholic steatohepatitis (53–55). One pre-clinical murine study inducing neuroinflammation by either systemic or intracerebroventricular lipopolysaccharide (LPS) showed effective reduction in neutrophil recruitment and reversal of nigrostriatal microglial activation by administering an analogous molecule, PXS-4681A (56). The same molecule demonstrated reduced neuroinflammation, dopaminergic neuroprotection and restored function in another murine study where LPS was administered into the substantia nigra (57). SSAO is an amine oxidase producing reactive oxygen species (ROS) in the vasculature and an adhesion protein which recruits leucocytes. MAO-B is an amine oxidase producing ROS in mitochondria of cells, including microglia and neurons, and an enzyme that catalyses degradation of central dopamine. Dual inhibition of SSAO and MAO-B could have potential benefit in central inflammatory disorders, especially with early dopaminergic involvement. This paper describes the design of the first drug trial of PXS-4728A in iRBD that aims at immune modulation to potentially provide an early, neuro-protective intervention in prodromal PD.

## Methods and analysis

The trial design and administrative details, including items from the World Health Organization Trial Registration Data Set, are summarised in Table 1.

**Table 1:** Structured summary of trial design and methods, including items from the world health organization trial registration data set.

WHO trial registration data set item	Detail
Primary registry and trial identifying number	ClinicalTrials.gov (NCT05904717) PXS4728A-IRBD-201
Date of registration in primary registry	2023-06-06
Secondary identifying numbers	Bellberry HREC 2023–03-289-A-5; Macquarie University HREC 520251690265568 EU CTA 22239/0006/001–0001 (MHRA); REC reference 23/EE/0256 (Cambridge South REC); ARSAC reference AA-7817;
Source(s) of monetary or material support	Parkinson’s UK Virtual Biotech programme, in partnership with the Parkinson’s Foundation
Primary sponsor	Syntara Limited Frenchs Forest, Australia
Secondary sponsor(s)	Not applicable
Contact for public queries	Corresponding author, Karolien Groenewald Nuffield Department of Clinical Neurosciences University of Oxford, UK karolien.groenewald@ndcn.ox.ac.uk
Contact for scientific queries	Corresponding author, Karolien Groenewald Nuffield Department of Clinical Neurosciences University of Oxford, UK karolien.groenewald@ndcn.ox.ac.uk
Public title	A study of oral PXS-4728A on brain microglial activation in people with isolated REM-sleep behaviour disorder (iRBD)
Scientific title	A Phase IIa, multicentre, double-blind, randomised, placebo-controlled, parallel-group study to assess the effect of 12 weeks’ treatment with oral PXS-4728A on microglia activation, as measured by positron emission tomography, in participants with isolated rapid eye movement sleep behaviour disorder
Countries of recruitment	Australia and United Kingdom
Health condition(s) or problem(s) studied	Isolated REM-sleep behaviour disorder (iRBD), a prodromal synucleinopathy
Intervention(s)	Active: PXS-4728A 15 mg oral, once daily for 12 weeks. Comparator: matched placebo, once daily for 12 weeks. Followed by a 12-week washout period.
Key inclusion and exclusion criteria	Inclusion: male or female aged 50 to 80 years with definitive PSG-confirmed iRBD, according to International Classification of Sleep Disorders criteria (ICSD-3). One or more features of parkinsonism, impaired olfaction and/or impaired colour vision (full criteria in Table 2). Exclusion: meets criteria for degenerative neurologic disorder, screening PET scan does not demonstrate robust [^11^C]-PK11195 striato-cortical signal (as visually assessed by the lead-imaging investigator; see Eligibility criteria), RBD associated with narcolepsy, dementia (MCI permitted), unstable medications (less than 4 weeks’ use) and untreated or severe obstructive sleep apnoea (full criteria in Table 3).
Study type	Purpose: Interventional/ treatment. Allocation: Randomised controlled trial, stratified by biological sex Intervention model: parallel group. Masking: double-blind (participants, investigators, study doctors, raters, biomarker analysts, including imaging, digital and laboratory personnel, and study statisticians). Type of endpoint: biomarker target engagement (TSPO ([^11^C]-PK11195) PET as a quantitative marker of microglial activation and of the biological activity of the study drug), drug safety and tolerability. Phase IIa.
Date of first enrolment	08 November 2023
Target sample size	40 participants (30 treatment, 10 placebo)
Recruitment status	Study completed
Primary outcome(s)	(1) Within-arm change in TSPO ([^11^C]-PK11195) PET binding across nigro-striato-cortical regions in the treatment arm from baseline to week 12. (2) Drug safety and tolerability over 12 weeks (safety laboratory tests, adverse events, treatment compliance, study completion).
Key secondary outcomes	Change in [^11^C]-PK11195 PET binding in additional cortical/subcortical regions and across the whole brain (baseline to week 12); change in cognition (MoCA) from baseline to weeks 12 and 24, in the treatment arm.
Ethics review	Approved UK: Cambridge South REC (23/EE/0256) Australia: Bellberry HREC (2023–03-289-A-5) and Macquarie University HREC (520251690265568).
Protocol version	Version 7.0 dated 04 November 2025. Version in effect at first enrolment: Version 3.0 dated 7 Jun 2023. Substantive amendments are summarised in Supplementary Table S1.
Protocol and statistical analysis plan availability	The full trial protocol (version 7.0, dated November 2025) and SAP are held by the sponsor, Syntara Ltd. Requests for access can be directed to the corresponding author and will be considered by the sponsor.
Completion date	17 June 2026
IPD sharing statement	There is currently no plan to share individual de-identified participant data externally. Syntara Limited, the study sponsor, is a small biotechnology company with limited resources to prepare and support a dataset for external use. However, requests for access to data underlying the findings reported in this publication will be considered on a case-by-case basis. Requests should be submitted to the corresponding author and include the research question, proposed analyses, and data required. Review will consider scientific merit, feasibility, participant consent and confidentiality, applicable legal and contractual obligations, commercially sensitive information, and the resources required to support access. Access is not guaranteed and, if approved, would be subject to appropriate de-identification and a data-use agreement.

## Overview

The PXS-4728A-IRBD trial is a randomised, double-blind, placebo-controlled, parallel-group study to investigate the biological activity and safety of an immune-modulating drug on neuroinflammation in iRBD. The trial will assess the effect of study drug (PXS-4728A) on neuroinflammation, by measuring change in TSPO PET binding in longitudinal TSPO ([^11^C]-PK11195) PET imaging, after a 12-week treatment period, in the treatment arm (within-arm). Forty participants will be randomised at a ratio of 3:1 to receive treatment (*n* = 30) or placebo (*n* = 10). Clinical assessment will be performed at screening, baseline, week 4, week 12 (end of treatment) and repeated after a 12-week washout period, week 24 (end of study).

In addition to drug effect, as measured with TSPO ([^11^C]-PK11195) PET, drug safety and exploratory end points will also be captured. Exploratory end points will measure change in clinical scales of prodromal symptoms (cognitive, mood, motor and other non-motor, such as dream enactment), biological markers from collected blood and CSF, and digital biomarkers (actigraphy, motor and cognitive). The trial timeline is illustrated in Figure 1.

**Figure 1:**
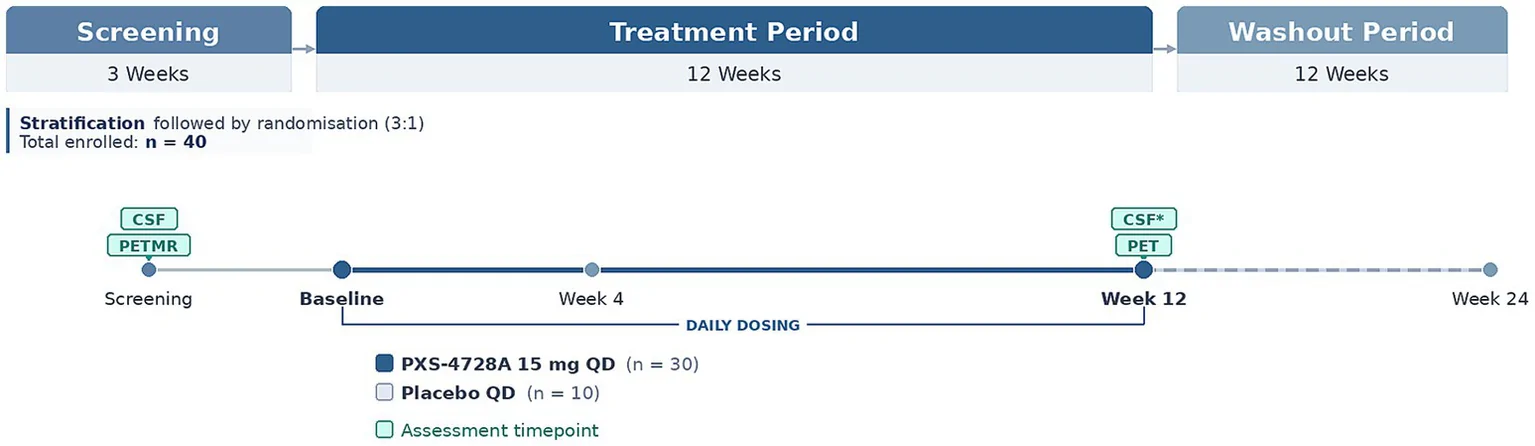
Overview of trial timeline. QD, once daily.

## Patient and public involvement

As part of the application for Parkinson’s UK Virtual Biotech Initiative funding, the trial was reviewed by four grant reviewers, including two lay members of the Virtual Biotech Involvement Group. The Oxford Parkinson’s Disease Centre (OPDC) has formed an iRBD participant advisory group for feedback and input regarding current and future research attempts.

## Participant identification

Participants will be recruited in parallel from two sites, Macquarie University in Sydney, Australia and the OPDC’s Discovery Cohort, University of Oxford in the United Kingdom. Each site is targeted to recruit 20 participants (*n* = 40 in total).

Australian participants will be identified from an ongoing prospective, longitudinal study phenotyping patients with polysomnography (PSG) confirmed iRBD (Macquarie University HREC #16842) where participants are evaluated annually. Participants were recruited through a combination of online radio promotions, targeted website advertising, and referrals from relevant specialist services. UK participants will be identified from the OPDC’s longitudinal Discovery cohort’s iRBD arm (South Central – Oxford A Research Ethics Committee IRAS number 188167). These participants have PSG confirmed iRBD and are followed up annually with extensive clinical, digital, imaging and wet-biomarker testing. Those who have consented to be contacted about other research studies will be pre-screened according to the inclusion and exclusion criteria, using the existing data base. Participants will receive an electronic copy of the participant information sheet and followed up telephonically to review any questions and to discuss their willingness to participate. They will then be booked for a screening visit, where written informed consent is obtained before continuing with study activities to establish trial eligibility.

## Eligibility criteria

Screened participants are deemed eligible once they fulfil inclusion (Table 2) and exclusion criteria (Table 3). This includes review of their symptomatology, medical and surgical histories, prior and concomitant medications, as well as a physical examination, electrocardiogram, baseline safety blood testing and baseline imaging, including [^11^C]-PK11195 PET and structural MRI.

**Table 2:** Inclusion criteria (Protocol V3.0—minor updates made in subsequent protocol versions).

Exclusion criteria	
1	Diagnosis of Parkinson’s disease, dementia with Lewy bodies, multiple system atrophy, or any other neurodegenerative parkinsonian disorder.
2	Secondary RBD (i.e., RBD attributable to narcolepsy, medication use, or other identifiable cause).
3	Montreal Cognitive Assessment (MoCA) score <23 at Screening.
4	Current use of medications known to cause or exacerbate RBD symptoms (e.g., SSRIs, SNRIs, tricyclic antidepressants, beta-blockers) unless on a stable dose for ≥3 months prior to Screening with no planned changes.
5	Use of any investigational drug within 30 days or 5 half-lives (whichever is longer) prior to Screening.
6	Current or recent (within 6 months) use of MAO-B inhibitors (e.g., selegiline, rasagiline, safinamide).
7	Known hypersensitivity to PXS-4728A or any of its excipients.
8	History of significant psychiatric disorder (e.g., schizophrenia, bipolar disorder, major depressive disorder requiring hospitalisation) within the past 2 years.
9	Active suicidal ideation or history of suicide attempt.
10	History of significant head trauma with loss of consciousness >30 min or any neurological sequelae.
11	History of stroke or transient ischaemic attack (TIA) within the past 12 months.
12	Uncontrolled hypertension (systolic BP > 160 mmHg or diastolic BP > 100 mmHg) at Screening despite treatment.
13	Clinically significant cardiac disease, including NYHA Class III/IV heart failure, unstable angina, or myocardial infarction within the past 6 months.
14	QTcF interval >450 ms (males) or >470 ms (females) at Screening or Baseline.
15	History of clinically significant hepatic disease or ALT/AST > 2 × upper limit of normal (ULN) at Screening.
16	Estimated GFR < 45 mL/min/1.73 m^2^ at Screening (CKD-EPI formula).
17	History of malignancy within the past 5 years, except adequately treated basal cell or squamous cell skin cancer, or carcinoma *in situ* of the cervix.
18	Known HIV, hepatitis B surface antigen positive, or hepatitis C antibody positive with detectable viral load.
19	Current evidence of alcohol or substance use disorder as defined by DSM-5 criteria.
20	Pregnant or breastfeeding females.
21	Contraindication to MRI (e.g., ferromagnetic implants, severe claustrophobia).
22	Contraindication to PET scanning or known hypersensitivity to [^11^C]-PK11195 or related radioligands.
23	Contraindication to lumbar puncture (e.g., coagulopathy, local skin infection, raised intracranial pressure).
24	Any surgical or medical condition that, in the investigator’s judgement, could interfere with study drug absorption, distribution, metabolism, or excretion.
25	Participation in another clinical trial with an active intervention within 30 days of Screening.
26	Inability or unwillingness to undergo vPSG during the Screening period.
27	Presence of significant sleep disorder other than RBD (e.g., severe obstructive sleep apnoea with AHI > 30, untreated restless legs syndrome) that could confound assessments.
28	Use of clonazepam or melatonin for RBD symptom management unless on a stable dose for ≥4 weeks prior to Baseline with no planned changes during the study.
29	Body weight <50 kg at Screening.
30	Known poor metaboliser status for CYP2D6 based on prior genotyping (if available).
31	Blood donation of ≥500 mL within 56 days prior to Screening.
32	Current use of strong CYP3A4 inhibitors or inducers within 14 days or 5 half-lives prior to first dose.
33	History of bariatric surgery or other gastrointestinal surgery that could affect drug absorption.
34	Clinically significant abnormality on Screening 12-lead ECG as judged by the investigator.
35	Any condition that, in the opinion of the investigator, would compromise the safety of the participant or interfere with interpretation of study results.
36	Employees of the sponsor or study site, or their immediate family members.
37	Positive urine drug screen for substances of abuse at Screening (unless explained by a prescribed medication).
38	Inability to communicate or cooperate with the investigator due to language barriers, cognitive impairment, or other reasons.

GCP, Good Clinical Practice; iRBD, isolated REM-sleep behaviour disorder; PET, positron emission tomography; MRI, magnetic resonance imaging.

**Table 3:** Exclusion criteria (Protocol V3.0—minor updates made in subsequent protocol versions).

Inclusion criteria	
1	Males and females aged 40–80 years at time of consent.
2	A diagnosis of isolated/idiopathic RBD (iRBD) confirmed by video polysomnography (vPSG) meeting the International Classification of Sleep Disorders (ICSD) criteria.
3	RBD symptom duration ≥1 year prior to Screening.
4	Modified Hoehn and Yahr stage ≤1.
5	Capable of providing informed consent and complying with study procedures.
6	Body weight ≥50 kg and BMI within 18–38 kg/m^2^ at Screening.
7	Females must be post-menopausal (≥12 months amenorrhoea, confirmed by serum FSH) or surgically sterilised.
8	Males with female partners of childbearing potential must agree to use adequate contraception from Screening through 90 days after last dose.
9	Ability to undergo MRI and PET scanning.
10	Stable doses of any concomitant medications for ≥4 weeks prior to Baseline.
11	Willing and able to comply with scheduled visits, treatment plan, and study procedures for the full duration of the study.

RBD, REM-sleep behaviour disorder; PET, positron emission tomography; MRI, magnetic resonance imaging.

The requirement for a robust striato-cortical [^11^C]-PK11195 signal on the screening PET scan (Table 2, criterion 5; Table 3, criterion 2) is a biomarker enrichment criterion, visually assessed by the lead imaging-investigator. This criterion ensures that participants have measurable pre-treatment TSPO binding, against which the treatment effect could be assessed as described in the outcome measures. However, this does restrict the interpretability of the findings to individuals with iRBD and relatively elevated striato-cortical TSPO binding, i.e., trial findings may not generalise to those with iRBD without this finding (see Discussion).

## Outcome measures

Primary outcomes will assess the within-arm change in [^11^C]-PK11195 binding potential (BP_ND_) on TSPO PET across nigro-striato-cortical regions in the treatment arm from baseline to week 12 and assess drug safety and tolerability, following a single daily dose of PXS-4728A for 12 weeks, by analysing safety lab test reports, adverse events, treatment compliance and study completion rates.

Secondary outcomes will assess the within-arm change in [^11^C]-PK11195 BP_ND_ on TSPO PET in additional cortical and subcortical regions of interest in the treatment arm from baseline to week 12. To check for any additional or unexpected signals, such analysis will also be performed across the whole brain. Changes in cognitive performance, as measured by the Montreal Cognitive Assessment (MoCA), from baseline to week 12 and week 24 will also be assessed in the treatment arm. MoCA was designated a secondary rather than exploratory outcome because it was the prespecified measure of global cognition used to monitor for unintended cognitive effects of MAO-B/SSAO inhibition and is registered as a secondary outcome on ClinicalTrials.gov.

Exploratory outcomes are considered hypothesis-generating only and will be measured from baseline to week 12 (treatment period) and week 12 to week 24 (washout period) in the following items:

- Quality of Life as assessed by the 36-Item Short Form Health Survey (SF-36).
- Patient and Clinical Global Impression of Change (PGIC and CGIC).
- Movement Disorder Society’s Unified Parkinson’s Disease Rating Scale (MDS-UPDRS I-III).
- Dream enactment as measured by the RBDSQ.
- Smell and colour discrimination, as measured by the Sniffin’ Sticks and Farnsworth-Munsell 100 Hue Test.
- Digital biomarkers, including the OPDC Smartphone, actigraphy and online cognitive test battery [Cognitron, administered for UK participants only (58)].
- Blood and CSF biomarkers including inflammatory cytokines (Mesoscale), alpha-synuclein (monomeric, aggregated forms using seed amplification assays and exosome associated), neurofilament light (SIMOA), PBMC mitochondrial and lysosomal function testing (Flow Cytometry), Apolipoprotein A1 and high sensitivity CRP.
- Between-arm differences (PXS-4728A versus placebo) in change in [^11^C]-PK11195 BP_ND_ in the primary and secondary regions of interest.

## Choice of comparator

There is currently no approved disease-modifying therapy for iRBD and a placebo comparator was therefore selected for this trial. Additionally, the placebo arm enables both the assessment of the safety and tolerability of PXS-4728A, as a co-primary outcome, and preserves double-blinding during study assessments.

Although this is double-blind, randomised, placebo-controlled trial, the primary imaging analysis evaluates change in [^11^C]-PK11195 BP_ND_ from baseline to week 12 within the treatment arm, and the trial is powered for this within-arm, rather than group-level, comparison (see Sample size calculation). This study was designed as an early-phase trial to establish whether PXS-4728A produces a measurable change in TSPO binding that would justify a larger placebo-controlled trial. A study design to detect between-group change would require a larger sample size for adequate statistical power. A potential within-arm [^11^C]-PK11195 BP_ND_ reduction measured in this study does not measure treatment effect, relative to placebo. It may also reflect measurement variability, regression towards the mean or time-related change. The between-group difference in change from baseline (PXS-4728A versus placebo) will therefore be estimated, with 95% confidence intervals, as an exploratory analysis to help interpret the within-arm result. Participants are randomised 3:1 to PXS-4728A or placebo, to ensure adequate statistical power for the primary imaging analysis, while retaining a control group adequate for the safety and blinding functions described above.

## Sample size calculation

The sample size calculation followed the approach used for a published Phase IIa randomised, placebo controlled, multicentre TSPO ([^11^C]-PBR28) PET study examining the effect of 8 weeks of treatment with AZD3241 (verdiperstat) on microglial activation in participants with PD (46).

In the AZD3241 (verdiperstat) trial, variability was estimated from a report by Gerhard et al. (38) describing eight PD participants who had undergone longitudinal [^11^C]-PK11195 PET imaging (at baseline and 24 months) (38). This showed a log relative change standard deviation (SD) in striatal binding potential from baseline of 0.553. Assuming the same SD, by recruiting 30 participants to the treatment arm, and assuming a 20% dropout rate (so 24 participants would complete the study), a two-sided paired t-test at α = 0.05 on log-transformed BP_ND_ has over 95% power to detect a 40% decrease and over 80% power to detect a 30% decrease in BP_ND_ from baseline in the treatment arm (standardised effect sizes of ~d = 0.92 and ~d = 0.65, respectively). Under these assumptions, the smallest reduction detectable with 80% power is approximately 28%.

The AZD3241 (verdipertsat) trial reported reductions of 13–16% in [^11^C]-PBR28 total distribution volume (V_T_) across nigrostriatal regions after 8 weeks, with effect sizes of 0.5–0.6 (46). Due to the generational difference in radioligands, associated signal-to-noise ratios and related outcome measures, these percentage changes are not directly comparable with BP_ND_ changes targeted in our study. The assumed reductions for our study correspond to the mentioned standardised effect sizes (~d = 0.65–0.92) of somewhat larger magnitude than those observed with AZD3241. The trial is not powered to detect smaller, but biologically potentially meaningful reductions. Non-significant primary results would therefore not exclude a smaller treatment effect and the estimated change with its 95% confidence interval will therefore be reported regardless of statistical significance.

## Trial procedures

### Clinical

Clinical scales assessing non-motor and motor symptoms will be performed at screening, baseline, week 4 (remote follow-up available), week 12 (end of treatment) and week 24 (end of study; see Table 4). Participants will continue their prescribed iRBD medication throughout the trial period.

**Table 4:** Schedule of assessments.

Schedule of assessments						
Assessment	Visit 1 Screening	Visit 2 Baseline	Visit 3 Week 4 Safety	Visit 4 Week 12 End of treatment	Visit 5 Week 24 End of study	*Ad Hoc*/ Early termination
Administrative						
Informed consent	✓					
Eligibility	✓	✓				
Demographics	✓					
Medical history^1^	✓	✓				
Prior and concomitant medication	✓	✓	✓	✓	✓	✓
Randomisation^5^		✓				
Dose administration^6^		✓	✓	✓		
Safety assessments						
Clinical examination^2^	✓	✓	✓	✓	✓	✓
Serum FSH^3^	✓					
Pregnancy test^4^	✓	✓		✓		✓
12-lead ECG^7^	✓	✓	✓	✓	✓	✓
Vital signs	✓	✓	✓	✓	✓	✓
Laboratory safety tests	✓	✓	✓	✓	✓	✓
Adverse events		✓	✓	✓	✓	✓
Telephone safety check^12^			✓			
Clinical and biomarker assessments						
Biological sampling	✓	✓	✓	✓	✓	✓
Smell and colour discrimination testing^8^	✓			✓	✓	✓
MDS UPDRS Parts I–III, OPDC Smartphone motor assessment^9^, Cognitron test battery^10^	✓	✓		✓	✓	✓
PGIC, CGIC, MoCA, SF-36, HADS, RBDSQ		✓		✓	✓	✓
Participant diary^11^		✓	✓	✓		
Imaging and procedures						
Axivity	✓			✓		
Lumbar puncture	✓			○ Opt.		
Structural MRI	✓					
TSPO ([^11^C]-PK11195) PET	✓			✓		

CGIC, Clinical Global Impression of Change; ECG, Electrocardiogram; EOS, End of study; ET, Early termination; FSH, Follicle-stimulating hormone; HADS, Hospital Anxiety and Depression Scale; MDS-UPDRS, Movement Disorder Society – Unified Parkinson’s Disease Rating Scale; MoCA, Montreal Cognitive Assessment; MRI, Magnetic resonance imaging; PGIC, Patient Global Impression of Change scale; RBDSQ, Rapid Eye Movement Sleep Behaviour Disorder Questionnaire; SF-36, 36-item short form survey; TSPO PET, Translocator Protein Positron Emission Tomography; WOCBP, Women of childbearing potential.

1 Medical history (including alcohol and smoking status), date of birth, age (calculated), sex, ethnicity, and race will be recorded at Screening.

2 A complete physical examination will be conducted at Screening and at the EOS/ET visit. A symptom-directed physical examination may be conducted at all other timepoints. Participant height will only be measured at Screening. Weight will be measured at all other timepoints.

3 Postmenopausal status will be confirmed through testing of FSH levels ≥ 40 IU/L at Screening for amenorrhoeic female participants.

4 For WOCBP, a serum pregnancy test will be performed at Screening and at all other times a urine pregnancy test will be conducted. If a urine test is positive, at the Investigator’s discretion, a serum test may be performed to confirm pregnancy status. If the serum test is negative, the participant may still be considered eligible.

5 Randomisation will occur on Day 1 any time prior to dosing.

6 Initial dosing will be conducted at the clinical site on Day 1 and all other dosing over the 12-week period will be conducted at home, except for the visits at Week 4 and Week 12. Dosing at Week 4 and Week 12 will be conducted at the clinical site under clinical supervision.

7 12-lead ECG, vital signs, laboratory safety tests, and biological sampling should be conducted within 60 min prior to dosing at Baseline, Week 4, and Week 12.

8 Testing of smell and colour discrimination will make use of validated methods such as Sniffin’ Sticks (smell) and the Farnsworth-Munsell 100 Hue test (colour).

9 At all physical visits, participants will be requested to complete the test twice, once at the beginning and once at the end of the procedures.

10 For participants in Australia, the Cognitron test battery is voluntary and may be conducted at home, at the discretion of the participant.

11 At Baseline visit, participants will be provided with a participant diary that will be used to track participant dosing, as well as safety events and participant experiences. Entries will be reviewed by the Investigator or delegated site staff at Visit 3 and Visit 4 to monitor treatment safety, AEs and compliance.

12 In the event that a participant cannot attend the clinical site at Week 4, a follow-up safety phone call may be conducted.

Non-motor symptom assessments include smell and colour discrimination testing (Sniffin’ Sticks, Farnsworth-Munsell 100 Hue Test), MDS-UPDRS non-motor symptom rating scales (Part I and II), cognitive (MoCA) and mood rating scales (HADS, PHQ-9, C-SSRS), QoL rating scales (SF-36) and RBD severity rating (RBDSQ). Motor symptoms will be assessed using the MDS-UPDRS Part III rating scale.

Safety tests include vital signs, ECG, blood and urine testing performed within an hour of in-clinic drug administration.

### Imaging

[^11^C]-PK11195 PET imaging will be used to assess change in TSPO binding from screening to week 12 (end of treatment).

Scanning for UK participants is conducted at the Wolfson Brain Imaging Centre (WBIC), Cambridge on a GE SIGNA PET/MRI scanner, where the radiotracer is produced at the WBIC Radiopharmaceutical Unit. Scanning for the Australian participants is conducted at Austin Health, Melbourne on a Philips Gemini TF64 PET/CT with radiotracer production at the same site. Because this is a PET/CT system, a separate baseline structural MRI is acquired for Australian participants at Macquarie University, Sydney.

Each participant is scanned on the same scanner, with the same acquisition and reconstruction protocol at screening and week 12. The within-participant change is therefore not affected by between-site scanner differences. Images were reconstructed using each vendor’s time-of-flight ordered-subset expectation-maximisation algorithm: VUE Point FX at WBIC (6 iterations, 16 subsets, no additional post-reconstruction filter applied), LOR-RAMLA at Austin Health. Attenuation correction was MRI-based at WBIC, using a derived measured attenuation map, and CT-based at Austin Health.

A target activity of 500 MBq [^11^C]-PK11195, with a minimum activity of 3 MBq/kg, is injected via a peripheral venous cannula over 30 s, and PET emission data is acquired for 60 min postinjection in 17-time frames.

All image processing and kinetic modelling are performed centrally, using the same pipeline for data from both sites. Site will be included as a covariate in exploratory statistical modelling (see Data analysis). Dynamic PET images will be corrected for motion, co-registered to the subject’s T1-weighted MRI, and processed to produce regional and voxel-wise non-displaceable binding potential (BP_ND_) estimates as a metric of [^11^C]-PK11195 binding to TSPO. BP_ND_ is a dimensionless number expressing the distribution volume ratio of specifically bound radioligand (V_S_) to that of non-displaceable radioligand (V_ND_) (59).

Quantification of BP_ND_ will be performed using the simplified reference tissue model, following the Cambridge group’s implementation (60, 61). Because no true reference region exists for [^11^C]-PK11195 given that TSPO is expressed throughout the brain and specific binding can occur in any anatomically defined region, a pseudo-reference input function will be derived using the supervised cluster analysis approach described by Yaqub et al. (60). This method identifies grey matter voxels whose kinetic behaviour matches a pre-defined low binding tissue class determined from healthy control subjects, thereby reducing contamination from voxels with specific TSPO binding that would otherwise bias anatomically defined reference regions.

Anatomical regions of interest will be defined using the Hammersmith atlas for automated anatomical labelling (n30r83) (62). Atlas regions will be warped from standard space to each participant’s native MRI space using nonlinear registration and then applied to co-registered PET images. Regional BP_ND_ values will be extracted for relevant predefined regions of interest (ROIs). Changes in [^11^C]-PK11195 BP_ND_ will be analysed in predefined ROIs from baseline to week 12 in the treatment group. The primary analysis comprises four separate analyses, one for each of the substantia nigra, caudate, putamen and a composite occipital cortex ROI (formed from the lingual gyrus, cuneus and lateral occipital regions of the atlas, and analysed as a single region); these are not combined into a composite or multivariate endpoint. As the aim of this early-phase study is to characterise preliminary biological activity and safety, no formal adjustment for multiplicity is planned. Results will be interpreted with this in mind, with emphasis on effect estimates, 95% confidence intervals and the consistency of effects across regions rather than on individual *p*-values. Secondary analysis ROIs will include all other cortical, deep and posterior fossa regions and subregions, as well as a total grey matter value. A voxel-wise analysis will also be performed to identify any additional clusters of reduced [^11^C]-PK11195 BP_ND_ from baseline to week 12 in the treatment group. A voxel-wise analysis will also be performed to identify any additional clusters of reduced [^11^C]-PK11195 BP_ND_ from baseline to week 12 in the treatment group. Cluster-level family-wise error (FWE) correction at *p* < 0.05, peak *p*-value FWE corrected, uncorrected cluster-level *p*-values and cluster sizes (voxels) will be reported. Secondary regional analyses are supportive and will not be adjusted for multiplicity. Image processing and extraction of regional and voxel-wise BP_ND_ values are performed blind to treatment allocation. Statistical analysis of the imaging data is performed by the central statistical team after database lock and unblinding.

## Bio-sample collection and processing

### Plasma and PBMC

Eight millilitres of whole blood will be collected into sodium citrate CPT tubes for plasma and PBMC collection at each clinical visit. Tubes are centrifuged at 1800G for 20 min at room temperature. Plasma supernatant is retained, aliquoted and stored at −80 °C for subsequent analysis. The PBMC layer is isolated from the same tube, washed with DPBS and centrifuged for another 15 min at 300G at room temperature, and the pellet resuspended for cell count, viability, aliquoting and cryopreservation. Cryopreserved PBMCs are stored in vapour phase liquid nitrogen for subsequent analysis.

### Serum

Ten millilitres of whole blood will be collected into SST tubes for the collection of serum at each clinical visit. Blood is allowed to clot at room temperature for 10 min then tubes are centrifuged at 1300G for 10 min. Serum is retained with aliquots stored at −80 °C for subsequent analysis.

### Cerebrospinal fluid

Fifteen millilitres of CSF will be collected at screening and optionally at Week 12/ end of treatment following participant agreement. CSF is placed on wet ice and processed within 2 h of collection. CSF is centrifuged at 1300G for 5 min at 4 °C to pellet cells. CSF supernatant is then aliquoted and stored at −80 °C. The cell pellet is then resuspended in cryopreservation media prior to cryopreservation and storing in vapour phase liquid nitrogen for subsequent analysis. CSF collection at week 12 is optional, and the paired number of samples is therefore expected to be limited. Therefore, baseline CSF analyses will be exploratory and longitudinal results descriptive.

### Exploratory fluid biomarkers

Collected biosamples will be used to assess measures of inflammation including cytokines, TREM2, C-reactive protein, apolipoprotein A1 and alpha-synuclein (monomeric and fibrillar as assessed by seed amplification assay). Neurofilament light, an indicator of neurodegeneration, will be measured in plasma and available CSF. Mitochondrial reactive oxygen species will be measured in PBMCs.

## Digital biomarkers

### OPDC smartphone application

Smartphone recordings will be performed twice at screening, baseline, end-of-treatment and end-of-study visits. This application consists of seven motor tasks including: voice recording, standing, walking, finger tapping (both hands), reaction test, rest tremor recording (both hands) and postural tremor recording (both hands). The test takes approximately 10 min to complete, and data is uploaded to a secure cloud server for analysis. The OPDC Smartphone application has been validated as an objective, remote, motor assessment tool that correlates well with UPDRS III scores as well as DaT SPECT findings in manifest and prodromal Parkinson’s Disease (63).

### Actigraphy

Actigraphy recordings will be performed for seven consecutive days at screening and optionally at the end-of-treatment visits using AX6 sensors worn on one (dominant) or both wrists. Data is stored on the devices and then uploaded to a secure cloud server for further analysis. Validated and open-source machine learning tools, such as ActiTect (64) can be employed to isolate RBD events from actigraphy recordings, whereby this can be used to quantify and detect change from baseline to week 12. Because week 12 actigraphy is optional, the expected data collection for paired tests will likely be limited to UK-based participants, as this is frequently included in the Discovery longitudinal cohort’s routine schedule of activities (*n* = 20).

### Cognitron test battery

An automated, online cognitive assessment will be completed after each clinical visit (screening, baseline, end-of-treatment and end-of-study) but is optional for Australian participants. Longitudinal data sets are therefore expected to be limited to UK-based participants (*n* = 20). This test is a remote and unsupervised 20-min test battery of eight tasks assessing visuospatial ability, executive function, short-term memory, language, and motor reaction time. The Cognitron test battery has been shown to correlate to in-clinic, supervised MoCA assessment, with improved sensitivity to detect early, subtle cognitive changes in iRBD and manifest PD (58).

## Treatment allocation, blinding and safety monitoring

Participants will be randomised 3:1 to receive active or placebo using a randomisation list prepared by an independent (not otherwise involved in the study) biostatistician using statistical software. Randomisation will be stratified for biological sex using permuted blocks within strata (block size not disclosed to sites). Allocation is concealed through the randomisation module of the Viedoc EDC system, which assigns a treatment kit number only after eligibility has been confirmed; site staff have no access to the allocation list. PXS-4728A and placebo capsules are identical in appearance and packaging. Treatment is 15 mg of PXS-4728A (or placebo) as a daily oral dose. Drug safety monitoring will be conducted at week 4 and week 12, including clinical assessment of vital signs, 12-lead ECG, blood and urine testing within 1 h of drug administration. Laboratory safety testing will include blood counts and liver, pancreatic, thyroid and kidney functions as well as pregnancy testing where indicated. Participant diaries will be reviewed at baseline, week 4 and week 12 to assess treatment compliance and review participant entries for adverse events. In the case of a serious adverse event where knowledge of treatment allocation is considered essential for the participant’s care, the Investigator can perform emergency unblinding of that participant’s treatment assignment via the electronic database, which records the unblinding; the study drug may be interrupted, delayed, reduced or discontinued. Participants are also able to withdraw from the trial at any time.

Following the trial, participants can continue follow up as part of site-specific iRBD observational cohorts.

## Trial monitoring and oversight

The trial is conducted under appropriate monitoring and oversight, with governance provided by the study sponsor, clinical research organisation and investigators, and regular review of trial conduct, data quality, and participant safety in accordance with the approved protocol, study specific plans and applicable regulatory requirements. Trial safety and monitoring will be conducted by the Clinical Research Organisation (CRO), Novotech. Novotech will conduct monthly site monitoring visits and have access to site-specific source documentation and EDC data. The CRO will report to the Sponsor of this study, Syntara Limited. Any substantial amendment to the protocol will be documented, approved by the sponsor, and submitted by the CRO to the Medicines and Healthcare products Regulatory Agency and to the relevant research ethics committees in the United Kingdom and Australia for approval prior to implementation. Approved amendments will be communicated to all participating sites and principal investigators, who are responsible for implementing the revised protocol, upon local approval, and for ensuring that all site personnel are informed. The ClinicalTrials.gov record (NCT05904717) will be updated to reflect substantial amendments. Where an amendment affects the information provided to participants or the basis of their consent, a revised participant information sheet and consent form will be approved by the relevant ethics committee, and participants will be re-consented. Non-substantial amendments will be documented and notified in accordance with applicable regulatory requirements.

A safety review committee (SRC) consisting of the Syntara medical director, Syntara Project Manager, Novotech medical monitor(s), Novotech project manager and principal investigator(s) will conduct meetings at set trial time points to review participant retention, drug safety data and make recommendations. Serious adverse events are reported to the sponsor and CRO (Pharmacovigilance Team) within 24 h and to regulatory authorities and the relevant research ethics committees in accordance with local requirements. The decision to suspend or terminate the trial rests with the sponsor, Syntara Ltd., informed by the SRC.

## Protocol amendments and timing of publication

The trial began enrolment on 08 November 2023 under protocol Version 3.0, dated 7 June 2023. The current protocol is Version 7.0, dated 04 November 2025; substantive amendments made during the trial are summarised in Supplementary Table S1. The statistical analysis plan was finalised on 21 June 2026, revised 21 July 2026, before database lock and unblinding of the statistical team on 28 July 2026. The analyses described here are those prespecified in the protocol and statistical analysis plan and were not modified after access to unblinded accumulating or completed trial data. This protocol is published after trial completion to provide a citable account of prespecified design, outcomes and the analysis plan.

## Data analysis

Trial source data will be collected on a combination of paper source and electronic recordings. The centralised electronic data capture (EDC) system (Viedoc) will assign a trial ID to each participant before pseudonymised data is uploaded to electronic case report forms. The electronic database assigns specific access and capabilities to individual team members to ensure all data is accurate, safe and of appropriate quality. Patient-identifiable data (PID) will be stored on a Secure Data Hosting Service, hosted by the relevant sites (Universities of Macquarie, Oxford, Cambridge and Austin Health). All study data will be securely stored for a minimum of 15 years in accordance with Australian regulatory and ethical requirements. Participant consent will include permission to share, and store pseudonymised data and samples between sites as well as additional, optional consent for future use of the collected biomarker data.

The modified intention-to-treat (mITT) population is defined as participants who received at least one dose of investigational product (PXS-4728A or placebo) and who have a baseline and at least one post-baseline assessment. This population will be used to analyse non-safety endpoints according to randomised treatment. The safety population comprises of all participants who received any dose of the investigational product (PXS-4728A or placebo) and will be used to analyse safety outcomes, according to treatment actually received. The per-protocol (PP) population comprises mITT participants with a treatment compliance rate above 80% through week 12, with no major protocol deviations and will be used in supplementary analyses of the primary endpoints.

All statistical tests will be two-sided with α = 0.05. Endpoints will be summarised by treatment group and visit using the mean and standard deviation of observed values and of change from baseline to week 12 and week 24. The primary and secondary imaging analyses will be based on log-transformed BP_ND_ (consistent with the sample size calculation) or in the case of negative BP_ND_ values, log transformed (BP_ND_ + 1; equivalent to distribution volume ratio (DVR)); untransformed BP_ND_ will also be analysed as a robustness check. Analyses will use a paired t-test to assess change from baseline to week 12 in the PXS-4728A group, for each region of interest separately, with the mean change and 95% confidence interval reported. In exploratory analyses of the imaging endpoints, the between-arm difference in change from baseline to week 12 will be estimated using two-sample t-tests and, additionally, a linear (analysis of covariance) model with treatment group as a fixed effect and adjustment for baseline [^11^C]-PK11195 BP_ND_, age, biological sex and study site. Given the small placebo group (*n* = 10), these analyses are not powered for hypothesis testing, and the emphasis will be on effect estimates and 95% confidence intervals.

For clinical, fluid and digital biomarker endpoints, change from baseline to week 12 and week 24 will be assessed. Within-arm estimates of change over time and between-arm comparisons will be presented with 95% confidence intervals, standard errors and nominal *p*-values (from paired or two-sample t-tests, as appropriate). These results are considered hypothesis-generating, and nominal *p*-values will not be interpreted as confirmatory. Analyses of optional or site-restricted assessments (Week 12 CSF, end-of-treatment actigraphy and the Cognitron battery) will include participants with available data only, with the descriptive number contributing reported for each endpoint and the availability of data summarised by treatment group and site.

To minimise missing data participants discontinuing early from treatment will be encouraged to continue with trial assessments. Missing data are therefore expected mainly for individual assessments within a visit rather than for whole visits. Primary analyses will be based on observed data, which assumes that data are missing completely at random. The extent, timing and reasons for missing data will be tabulated by treatment group, and the plausibility of this assumption will be assessed by comparing baseline characteristics of participants with and without missing data. Sensitivity analyses using multiple imputation (under a missing-at-random assumption) will be conducted where the amount or pattern of missing data could plausibly affect interpretation of an endpoint, including where >5% of participants in the PXS-4728A group have missing data, where missingness appears imbalanced between treatment groups, or where it is associated with a baseline characteristic.

## Discussion

This protocol describes a novel trial of an immune-modulating treatment in a clinically enriched iRBD cohort, targeting a potentially reversible contributor to prodromal synuclein disease with an objective biomarker of neuroinflammation.

Prior TSPO PET reports, using first generation tracer [^11^C]-PK11195, established evidence for *in vivo*, longitudinal neuroinflammatory changes in iRBD, in which higher degrees of baseline [^11^C]-PK11195 binding were associated with a reduction in longitudinal dopaminergic function. This protocol therefore proposes the methods to measure, and target neuroinflammation, within a clinically enriched population in the prodrome of synuclein disease, prior to the onset of clinically established dopaminergic dysfunction and defined neurodegeneration.

The proposed trial utilises a modest sample size (*n* = 40, randomised 3:1) which provides adequate power for the within-arm primary imaging outcome (a 30–40% reduction in [^11^C]-PK11195 BP_ND_ from baseline to end of treatment in the PXS-4728A group), and not for between-arm comparisons. Safety and tolerability will be described in both groups. Although the use of [^11^C]-PK11195 has challenges, including the relatively low signal-to-noise ratio, it remains widely used, with sufficient existing reference data, and is not impacted by genetic variability in the TSPO gene (rs6971 polymorphism) and resultant differential binding as seen in second generation TSPO tracers. The sample size and trial length (12 weeks) may limit the analysis of between-arm clinical and imaging changes.

The principal limitation of this design is that the primary imaging analysis is a within-arm comparison. A reduction in [^11^C]-PK11195 binding in the PXS-4728A group could reflect test–retest variability of [^11^C]-PK11195 quantification, time-related change or regression towards the mean, rather than a treatment effect. A significant within-arm reduction will be interpreted as evidence of biological activity, warranting further evaluation of treatment effect. The exploratory comparison with the placebo group will help to contextualise within-arm changes, but the interpretation is limited due to the small sample size.

The eligibility criteria (PSG-confirmed iRBD, mild Parkinsonism, hyposmia and/or impaired colour discrimination, and a robust baseline [^11^C]-PK11195 signal) define an enriched population with markers of prodromal progression and elevated TSPO binding. This enrichment strategy increases the likelihood of detecting a change in TSPO binding, but participants may not be representative of the broader iRBD population, and the trial findings may not generalise to all people with iRBD.

This trial is designed to provide evidence of biological activity, as measured by TSPO PET binding, which may in turn inform future, longer and larger interventional trials in iRBD. The protocol may offer a template for future disease-modifying trials in prodromal synuclein disease.

## Strengths and limitations of this study

- This is the first randomised, double-blind, placebo-controlled trial of an immune-modulating agent in isolated REM-sleep behaviour disorder, a prodromal synucleinopathy.
- Neuroinflammation is assessed objectively using TSPO ([^11^C]-PK11195) PET as a quantitative marker of microglial activation and of the biological activity of the study drug.
- Participants are recruited from established, longitudinally phenotyped, polysomnography-confirmed iRBD cohorts, supporting diagnostic certainty with enhanced clinical characterisation.
- The modest sample size (*n* = 40) and 12-week treatment window may limit power to detect smaller or more gradual effects on neuroinflammation.
- The use of [^11^C]-PK11195, a first-generation TSPO radioligand, minimises non-affinity binding seen in second generation tracers due to rs6971 polymorphisms. However, its relatively low signal-to-noise ratio may limit sensitivity to detect smaller changes in microglial activation compared to second generation radioligands.
- The study is statistically powered for the primary imaging analysis as a within-arm comparison in the treatment group only. Between-arm differences will be treated as exploratory.
- Week 12 CSF collection and some digital biomarker assessments were treated as optional for participants and sites, this reduces the sample size and interpretability of some exploratory endpoints.
