# Supplementary material for: The protocol for a phase 2A, multi centre, double-blind, randomized, placebo-controlled, parallel-group study to assess the effect of 12 weeks treatment with oral PXS-4728A on microglia activation, as measured by positron emission tomography, in participants with isolated rapid eye movement sleep behaviour disorder
Source: Front Neurol. 2026 Oct 28;17:1951452. doi: 10.3389/fneur.2026.1951452 (PMC13753547; doi:10.3389/fneur.2026.1951452)
Supplement: Supplementary file 1 [file Table_1.docx]

| **Supplementary Table 1: Protocol Versions and Amendments** | | |
| --- | --- | --- |
| **Protocol version** | **Date** | **Main changes from previous version** |
| **V1.0** | 19-Dec-2022 | N/A initial version |
| **V2.0** | 28-Mar-2023 | Addition of MoCA as secondary objective. |
| **V3.0** *(version current when enrolment began)* | 7-Jun-2023 | Increased screening period from 7 to 21 days to allow sufficient time to complete all required assessments. |
| **V4.0** | 19-Jan-2024 | Expanded exclusion criteria to include suicidal ideation as well as suicide attempts. Addition of Columbia-Suicide Severity Rating Scale (C-SSRS) as an additional safety monitoring measure. |
| **V5.0** | 30-Apr-2024 | Clarification that structural MRI and TSPO PET will be centrally reviewed. Change of sponsor name from Pharmaxis Ltd to Syntara Limited. |
| **V6.0** | 18-Feb-2025 | Minor clarifications to inclusion and exclusion criteria. |
| **V7.0** *(current version at completion, and when unblinding for analysis occurred on 28-Jul-2026)* | 20-Oct-2025 | Minor administrative updates to study personnel. Update to expected completion date. |

| **Supplementary Table 2: Statistical Analysis Plan Versions** | | |
| --- | --- | --- |
| **Version** | **Date** | **Main changes from previous version** |
| **V1.0** | 21-Jun-2026 | N/A initial version containing more details of analyses specified in protocol. |
| **V2.0** *(current)* | 21-Jul-2026 | Additional sensitivity and exploratory analyses added. (No changes to primary analysis methods compared to V1.0) |
